# The Multicenter Study of a New Assay for Simultaneous Detection of Multiple Anti-Aminoacyl-tRNA Synthetases in Myositis and Interstitial Pneumonia

**DOI:** 10.1371/journal.pone.0085062

**Published:** 2014-01-14

**Authors:** Ran Nakashima, Yoshitaka Imura, Yuji Hosono, Minae Seto, Akihiro Murakami, Kizuku Watanabe, Tomohiro Handa, Michiaki Mishima, Michito Hirakata, Tsutomu Takeuchi, Keishi Fujio, Kazuhiko Yamamoto, Hitoshi Kohsaka, Yoshinari Takasaki, Noriyuki Enomoto, Takafumi Suda, Kingo Chida, Shu Hisata, Toshihiro Nukiwa, Tsuneyo Mimori

**Affiliations:** 1 Department of Rheumatology and Clinical Immunology, Graduate School of Medicine, Kyoto University, Kyoto, Japan; 2 Medical & Biological Laboratories Co., Ltd., Nagoya, Japan; 3 Department of Respiratory Medicine, Graduate School of Medicine, Kyoto University, Kyoto, Japan; 4 Medical Education Center/Department of Internal Medicine, Keio University School of Medicine, Tokyo, Japan; 5 Division of Rheumatology, Department of Internal Medicine, Keio University School of Medicine, Tokyo, Japan; 6 Department of Allergy and Rheumatology, Graduate School of Medicine, The University of Tokyo, Tokyo, Japan; 7 Department of Medicine and Rheumatology, Graduate School of Medical and Dental Sciences, Tokyo Medical and Dental University, Tokyo, Japan; 8 Department of Internal Medicine and Rheumatology, Juntendo University School of Medicine, Tokyo, Japan; 9 Second Division, Department of Internal Medicine, Hamamatsu University School of Medicine, Hamamatsu, Japan; 10 Department of Respiratory Medicine, Tohoku University Graduate School of Medicine, Sendai, Japan; Keio University School of Medicine, Japan

## Abstract

Autoantibodies to aminoacyl-tRNA synthetases (ARSs) are useful in the diagnosis of idiopathic inflammatory myopathy (IIM) with interstitial pneumonia (IP). We developed an enzyme-linked immunosorbent assay (ELISA) system using a mixture of recombinant ARS antigens and tested its utility in a multicenter study. **Methods:** We prepared six recombinant ARSs: GST-Jo-1, His-PL-12, His-EJ and GST-KS expressed in *Escherichia coli*, and His-PL-7 and His-OJ expressed in Hi-5 cells. After confirming their antigenic activity, with the exception of His-OJ, we developed our ELISA system in which the five recombinant ARSs (without His-OJ) were mixed. Efficiency was confirmed using the sera from 526 Japanese patients with connective tissue disease (CTD) (IIM n = 250, systemic lupus erythematosus n = 91, systemic sclerosis n = 70, rheumatoid arthritis n = 75, Sjögren’s syndrome n = 27 and other diseases n = 13), 168 with idiopathic interstitial pneumonia (IIP) and 30 healthy controls collected from eight institutes. IIPs were classified into two groups; idiopathic pulmonary fibrosis (IPF) (n = 38) and non-IPF (n = 130). Results were compared with those of RNA immunoprecipitation. **Results:** Sensitivity and specificity of the ELISA were 97.1% and 99.8%, respectively when compared with the RNA immunoprecipitation assay. Anti-ARS antibodies were detected in 30.8% of IIM, 2.5% of non-myositis CTD, and 10.7% of IIP (5.3% of IPF and 12.3% of non-IPF). Anti-ARS-positive non-IPF patients were younger and more frequently treated with glucocorticoids and/or immunosuppressants than anti-ARS-negative patients. **Conclusion:** A newly established ELISA detected anti-ARS antibodies as efficiently as RNA immunoprecipitation. This system will enable easier and wider use in the detection of anti-ARS antibodies in patients with IIM and IIP.

## Introduction

A number of autoantibodies can be detected in sera from patients with idiopathic inflammatory myopathy (IIM), some of which are specific to IIM (known as myositis-specific autoantibodies: MSAs). Detection of these autoantibodies is closely associated with IIM clinical manifestations [1], [2].

Among MSAs, autoantibodies against aminoacyl-tRNA synthetases (ARSs) are the most frequently detected in adult IIM patients. To date, eight anti-ARS antibodies have been described. Anti-Jo-1 (histidyl-tRNA synthetase) [3], [4] is the most common, occurring in approximately 20% of IIM patients [2], [5]. Anti-PL-7 (threonyl) [6], anti-PL-12 (alanyl) [7], [8], and anti-EJ (glycyl) [9] occur in ∼3–4%, and anti-OJ (isoleucyl) [10] and anti-KS (asparaginyl) [11] occur in < 2% of IIM patients. Anti-tyrosyl- and anti-phenylalanyl-tRNA synthetases were also reported in one case each [12], [13]. Patients with anti-ARSs show a spectrum of common clinical manifestations known as anti-synthetase syndrome (ASS), including myositis, interstitial pneumonia (IP), non-erosive arthritis, fever, Raynaud’s phenomenon, and mechanic’s hands. Of note, the prevalence of IP in anti-ARS-positive patients is as high as 75–95% and IP sometimes precedes myositis [1], [14], [15]. Yoshifuji *et al.* reported that anti-ARS-positive patients with IP respond better to initial corticosteroid therapy but suffer from a significantly higher recurrence than anti-ARS-negative patients [1]. Therefore, anti-ARS antibodies are useful not only in diagnosing IIM but also in predicting late-onset myopathy in IP-proceeding patients and the clinical course of IP in myositis.

Currently, anti-ARS antibodies are detected using an enzyme-linked immunosorbent assay (ELISA), immunodiffusion or immunoprecipitation, but all of the antibodies are not routinely detected except for anti-Jo-1. To detect anti-ARS antibodies more readily, we established an ELISA system using a mixture of five recombinant ARS antigens: Jo-1, PL-7, PL-12, EJ, and KS. Our intention was to detect these autoantibodies simultaneously as “multiple anti-ARS antibodies”. This ELISA system that we developed could be used to detect not only anti-ARS-positive myositis patients but also anti-ARS-positive idiopathic interstitial pneumonia (IIP) patients.

## Materials and Methods

### Patients

Serum samples were obtained from 694 Japanese adult patients with connective tissue disease (CTD) and IIP who had been followed at eight University Hospitals in Japan and 30 healthy volunteers. Patient diagnoses included IIM (n = 250), systemic lupus erythematosus (SLE) (n = 91), systemic sclerosis (SSc) (n = 70), rheumatoid arthritis (RA) (n = 75), SS (n = 27), other diseases (n = 13), and IIP (n = 168). The diagnoses of IIM, SSc, SLE, and RA were made on the basis of corresponding criteria proposed by Bohan and Peter [16] or the American College of Rheumatology [17], [18], [19]. IIP was defined as IP of unknown cause in which a patient did not fulfill classification criteria for any specific CTD or vasculitis, or whose lung disease was potentially caused by a drug or occupational-environmental exposure [20]. Patients with IIP were classified into two groups; an idiopathic pulmonary fibrosis (IPF) (n = 38; 12 by histological diagnosis) group and a non-IPF (n = 130; according to the typical radiographic patterns of chest high-resolution computed tomography) group.

All patients and healthy volunteers gave their written informed consent to participate in this study prior to sample collection that was performed in accordance with the Declaration of Helsinki. This study was approved by the Ethics Committee of Kyoto University Graduate School and Faculty of Medicine (Approval number: E544) and also by institutional review boards of all participating centers (Table S1).

### Immunoprecipitation

The presence of anti-ARS antibodies was determined by RNA immunoprecipitation (RNA-IP) as previously described [21]. The immunoprecipitated RNA was resolved using urea-polyacrylamide gel electrophoresis and visualized using silver staining. Each anti-ARS antibody was identified according to its mobility and tRNA pattern compared with standard serum.

### Construction of expression plasmids for ARS-encoding cDNAs

For the expression and purification of recombinant proteins, full-length cDNAs of PL-12, EJ, PL-7, Jo-1, KS, and OJ (GenBank accession Numbers: D32050, U09587, NM_152295, AY995220, and BC001687, respectively) were first amplified using RT-PCR with HeLa total mRNA as a template. CDNAs for PL-12 and EJ were inserted into pET30a(+) (Novagen, Madison, WI, USA) and expressed as C-terminal His-tagged proteins. CDNAs for Jo-1 and KS were subcloned into pGEX4T-1 and pGEX6P-1 (GE Healthcare UK Ltd, Buckinghamshire, England), respectively, and expressed as N-terminal GST fusion proteins. CDNAs for PL-7 and OJ were engineered with a cMyc-epitope tag and His-tag sequence at their 3′ ends, and inserted into the pFastBacDual vector for baculovirus expression (Invitrogen, Carlsbad, CA, USA). Correct construction of plasmids was confirmed using DNA sequencing.

### Expression and purification of recombinant ARSs

*Expression and purification of His-tagged recombinant proteins*: PL-12 and EJ were expressed in *Escherichia coli* BL-21(DE3) codon plus RIL bacteria (Stratagene, La Jolla, CA, USA). Competent cells were transformed with the vectors and the cells were incubated on Luria-Bertani (LB) agar plates containing 50 µg/mL kanamycin for 15 h at 37°C. A single colony was cultured in LB liquid medium containing kanamycin at 37°C. Addition of 1 mM isopropyl-1-thio-β-D-galactopyranoside to the medium was used to induce expression of recombinant PL-12 and EJ proteins. After a 2-h incubation, cells were harvested using centrifugation and resuspended in ice-cold phosphate buffered saline (PBS) at pH 7.5. The cells were sonicated and soluble cell lysates containing the His-tagged recombinant proteins were separated using centrifugation.

PL-7 and OJ were expressed in baculovirus-infected Hi-5 cells. Each of the expression vectors was transfected into SF-9 cells using Cellfectin (Invitrogen), and the baculovirus stock was prepared from the transfectant culture supernatant. Hi-5 cells infected with baculovirus were incubated for 72 h at 26°C and were harvested using centrifugation, and soluble cell lysates containing recombinant proteins were prepared as described above.

Soluble His-tagged recombinant ARSs were purified using immobilized metal ion affinity chromatography. Cell extracts were applied to TALON® Metal Affinity Resin columns (Clontech, Palo Alto, CA, USA), and the columns were washed with PBS containing 10 mM imidazole. Purified PL-12, EJ, PL-7, and OJ were eluted with PBS containing 50 mM imidazole.

*Expression and purification of recombinant GST-ARS fusion proteins*: Jo-1 and KS were also expressed in *E. coli* BL-21(DE3) codon plus RIL bacteria in the presence of ampicillin. Transformation, cultivation, induction, and extraction of soluble cell proteins were performed as described for PL-12 and EJ proteins. Soluble GST-Jo-1 and GST-KS fusion proteins were purified on Glutathione Sepharose 4B columns (GE Healthcare UK Ltd.) and eluted with Tris-HCl (pH 8.0) containing 15 mM GSH.

### Immunoblotting of recombinant antigens

Purified recombinant ARS antigens were subjected to sodium dodecyl sulfate polyacrylamide gel electrophoresis (SDS-PAGE) and transferred to a polyvinylidene difluoride (PVDF) membrane as described by Towbin *et al.* [22] with minor modifications. After blocking with 5% skimmed milk, the membrane was incubated for 60 min with serum diluted 1∶100 and then incubated for 60 min with a 1∶1000 dilution of goat anti-human IgG conjugated to peroxidase (Code No. 208, MBL, Nagoya, Japan). Immunoreactive bands were detected using the Western Blot Detection System WEST-one (iNtRON Biotechnology, Gyeonggi-do, Korea).

### ELISA

For detection of each ARS autoantibody, purified recombinant ARSs were individually coated on 96-well microtiter plates (Maxisorp; Nunc, Rochester, NY, USA). PL-12, EJ, PL-7, and Jo-1 were diluted in PBS to a final concentration of 2.5 µg/mL, and KS to 5.0 µg/mL. Each diluent was added at 100 µL/well and incubated overnight at 4°C. The plates were washed twice with PBS, and blocked with PBS containing 1% bovine serum albumin (BSA) and 5% sucrose overnight at 4°C. Sera from patients and normal healthy donors were diluted 1∶100 in PBS containing 0.15% Tween 20 (PBS-T), 1% casein enzymatic hydrolysate, and 0.2 mg/mL *E. coli* extract, and 100 µL was applied to each well. After incubation for 60 min at room temperature (RT), the wells were washed four times with PBS-T. Goat anti-human IgG conjugated to peroxidase (Code No. 208, MBL) was diluted 1∶7000 in 20 mM HEPES, 135 mM NaCl, 1% BSA, and 0.1% hydroxyphenylacetic acid (peroxidase stabilizer), and 100 µL was added to each well. After incubation for 60 min at RT, the wells were washed four times with PBS-T, and 3,3’,5,5’-tetramethylbenzidine substrate was then added. After a 30-min incubation at RT, the reaction was stopped by adding 100 µL of 0.25 N sulfuric acid and absorbance was read at 450 nm (A_450_).

For simultaneous detection of five ARS autoantibodies, purified recombinant ARSs were diluted and mixed together in PBS and coated on plates. The final concentrations of PL-12, EJ, PL-7, Jo-1, and KS were 1.25 µg/mL, 0.63 µg/mL, 1.25 µg/mL, 0.63 µg/mL, and 2.5 µg/mL, respectively. The total protein concentration of the mixture was 6.25 µg/mL. ELISA plate preparation and assays were performed as described above. Conversion from A_450_ to a unit value (U/mL) was calculated using the following formula:
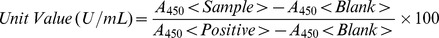



A_450_<Positive> is the absorbance for an anti-Jo-1-positive patient serum that corresponds to a 100 U/mL value. A_450_<Blank> is the background absorbance of buffer that does not contain serum. A_450_<Sample> is the absorbance of a tested serum. The cutoff point was defined at 25 U/mL based on the analysis of the receiver operating characteristic curve in this multicenter study.

### Statistical analysis

Statistical analyses were performed using StatView version 5.0 software. Clinical information of anti-ARS-negative and positive non-IPF patients was compared using the two-sample t-test or the Fisher’s exact test.

## Results

### Autoantigen preparation

**Figure 1:**
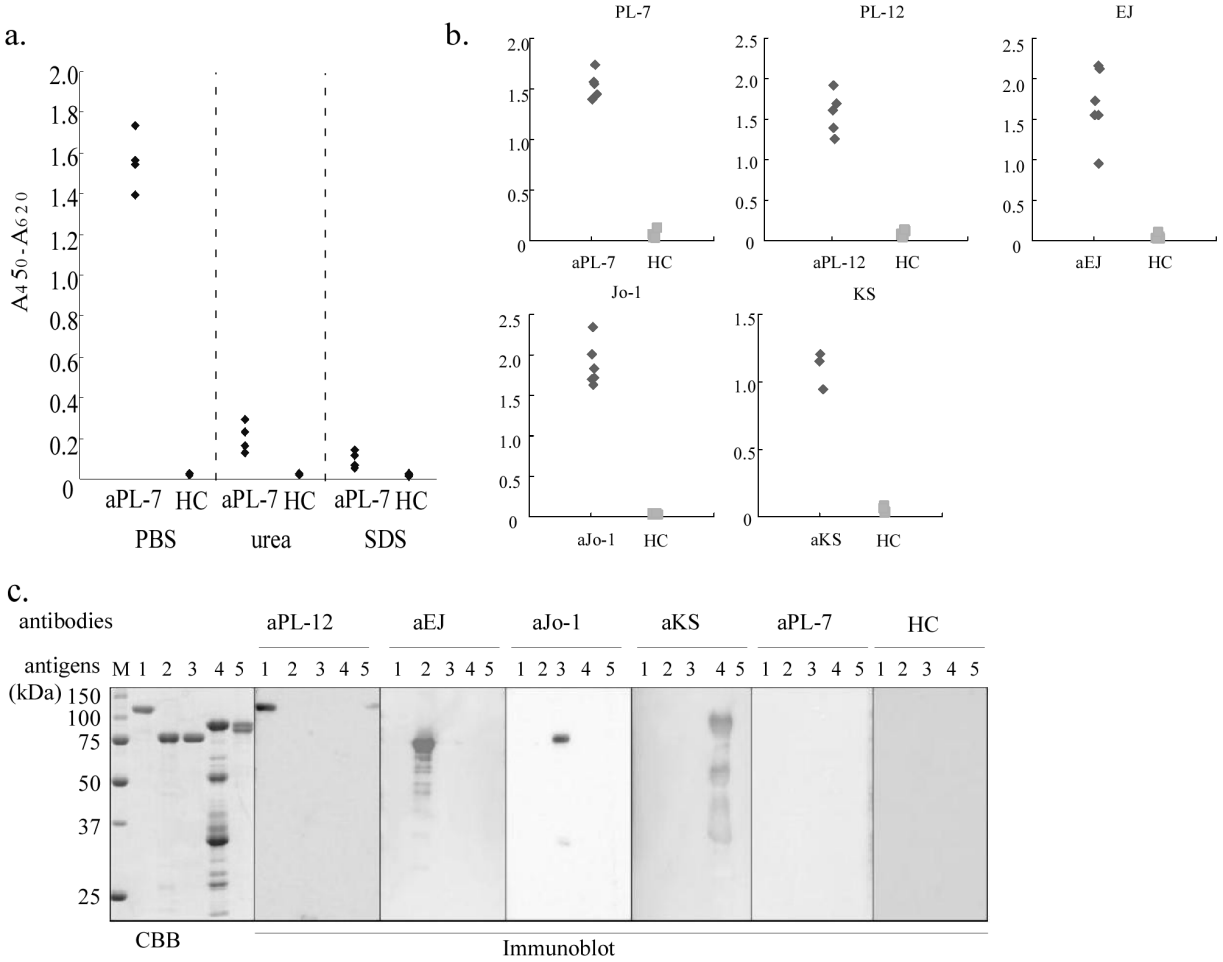
Antigenic activity of recombinant autoantigens a. Antigenic activity of PL-7 in various conditions. Left, purified recombinant PL-7 was eluted and diluted in PBS and coated on ELISA plates. Middle and Right, purified recombinant PL-7 was eluted in PBS and diluted in 8M urea and 2 × SDS sample buffer, respectively, and then coated onto ELISA plates. b. Five recombinant ARS antigens (His-PL-12, His-EJ, GST-Jo-1, GST-KS, and His-PL-7) were prepared as soluble polypeptides in PBS and their antigenic activity was tested in an ELISA using sera from five patients each containing corresponding autoantibodies (only GST-KS was tested using sera from three patients). Six healthy controls were used in each ELISA. c. Purified recombinant ARS antigens were electrophoresed on SDS-PAGE and transferred to a PVDF membrane followed by immunoblot analysis. CBB; Coomassie Brilliant Blue staining of gels, M; molecular weight marker, HC; healthy control, Lane 1; His-PL-12, Lane 2; His-EJ, Lane 3; GST-Jo-1, Lane 4; GST-KS and Lane 5; His-PL-7.

We first prepared six recombinant His-tagged ARS antigens, which were all expressed in *E. coli*. Immunoblot analysis showed that four of them, Jo-1, PL-12, EJ, and KS, were identified by their corresponding autoantibodies as well as by using an ELISA, whereas PL-7 and OJ reacted weakly with their corresponding autoantibodies (data not shown). Because we hypothesized that poor antigenic activity of recombinant PL-7 and OJ was due to a lack of posttranslational modification or proper structural folding, we prepared both fusion proteins expressed in eukaryotic Hi-5 cells using the baculovirus system. We confirmed antigenic activity of the new recombinant PL-7 using an ELISA (Fig. 1a) but the activity was lost when examined using immunoblotting (Fig. 1c). Recombinant PL-7, denatured using urea or SDS, had weaker antigenic activity than non-denatured PL-7, showing that the 3-dimensional protein structure played an important role in the reaction between the threonyl-tRNA synthetase and the anti-PL-7 antibody (Fig. 1a). Because of this antigenic characteristic of PL-7, we decided to prepare other recombinant ARSs, without denaturing reagents, as soluble polypeptides in PBS. Because His-Jo-1 and His-KS were insoluble, they were expressed as GST-recombinant proteins. ELISA revealed that the five newly prepared ARS antigens, His-PL-12, His-EJ, GST-Jo-1, GST-KS, and His-PL-7, displayed suitable antigenic reactivity. Immunoblotting also showed that four of the five ARS antigens, except for His-PL-7, had sufficient antigenic activity (Fig. 1b and c).

The recombinant OJ expressed in Hi-5 cells had weak antigenic activity, as confirmed using both immunoblotting and an ELISA (data not shown), suggesting that it is difficult to prepare a recombinant OJ as a single polypeptide that retains antigenic activity.

### Establishing an ELISA system for simultaneous detection of five ARS antibodies

To detect multiple ARS antibodies simultaneously, we developed an ELISA system using a mixture of the five recombinant ARSs except for OJ. We tested a variety of antigen mixtures to estimate the most appropriate ratio and concentration to use, and we found that anti-ARS-positive sera showed reactivity with all five different ARSs with the highest sensitivity and specificity occurring at antigen concentrations of 0.63, 1.25, 1.25, 0.63, and 2.5 μg/mL (6.25 μg/mL in total) for histidyl-, threonyl-, alanyl-, glycyl-, and asparaginyl-tRNA synthetases, respectively. To assess potential cross-reactivity, we compared the absorbance values (A_450_) obtained using an ELISA on every single recombinant ARS with those obtained with the new ELISA using the ARS mixture. When tested using a single-peptide-ELISA, each of the five anti-ARS antibodies showed reactivity with only its corresponding autoantigen. Samples positive for anti-PL-7, PL-12, or KS antibodies showed higher A_450_ values with the new mixed-peptide-ELISA than with the single-peptide ELISA, whereas the samples positive for anti-Jo-1 or EJ antibodies showed no significant difference in A_450_ values obtained with the two ELISAs. Such differences in A_450_ values may be due to different peptide-coating efficiencies because the total peptide concentration was higher in the mixed-peptide-ELISA than in the single-peptide ELISA (data not shown).

### Clinical significance of anti-ARS ELISA in CTD

**Figure 2:**
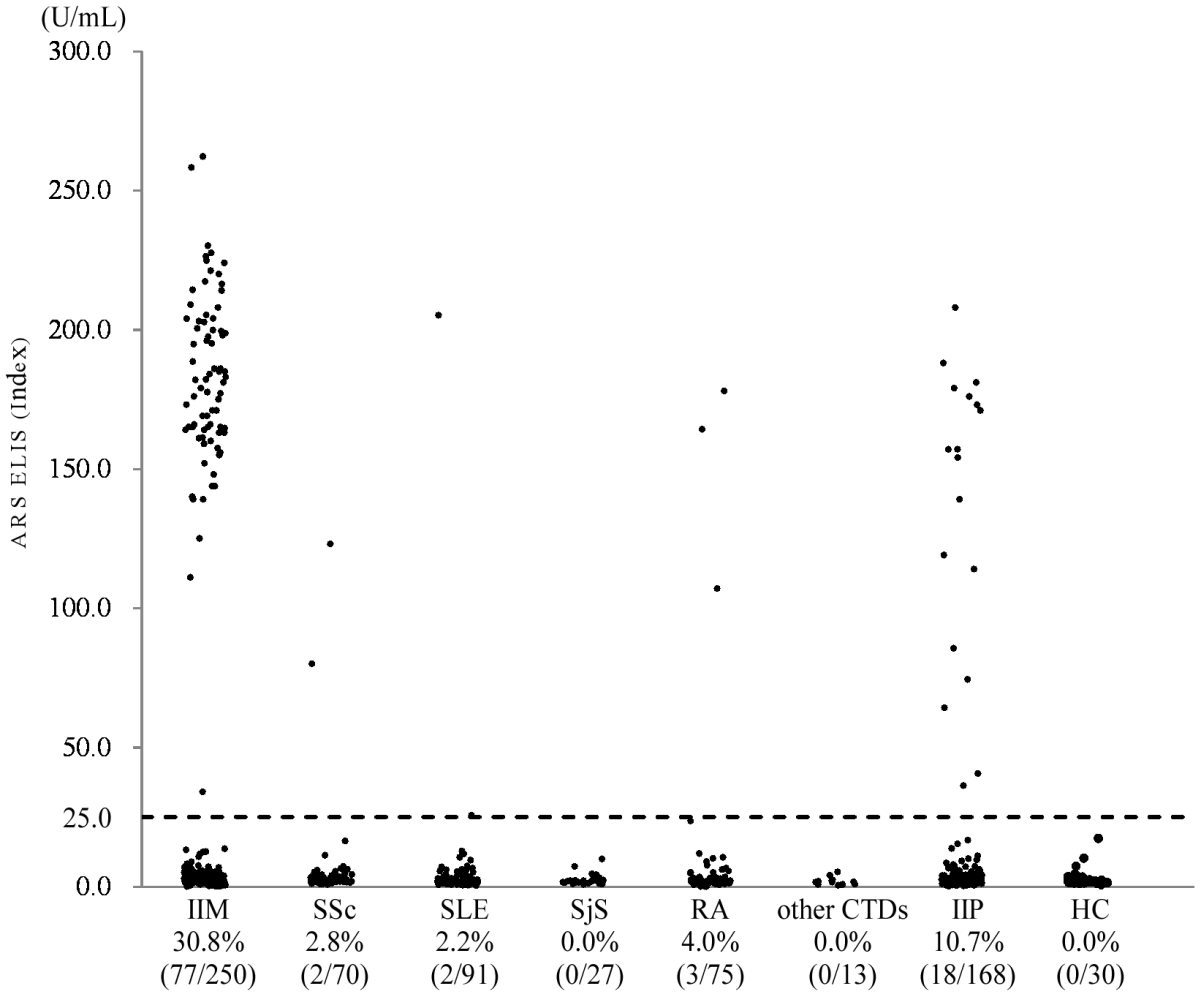
Confirmation of the efficiency of the ELISA system. Using the ELISA system, ARS antibodies were measured in 694 serum samples from patients with various CTDs and IIP, and 30 serum samples from healthy controls. The cutoff value (25 U/mL) is indicated by a horizontal dotted line.

To confirm the efficiency of this newly established ELISA, we screened a total of 694 serum samples from patients with various CTDs and IIP, and 30 healthy controls. The results were compared between the ELISA and the RNA-IP assay (Fig. 2). A total of 102 samples were positive for anti-ARS antibodies using the ELISA and all of them, except for one, were identified to have any anti-ARS, other than anti-OJ, using the RNA-IP assay (Table 1). The sensitivity and specificity of the new ELISA in the detection of anti-ARS antibodies (including anti-OJ) compared with the RNA-IP technique were 97.1% and 99.8%, respectively (Table 1). Anti-ARS antibodies were detected in 30.8% (77/250) of IIM and 2.5% (7/276) of other CTDs (Table 2). None of the healthy controls were positive (Fig. 2). In IIM, 30.8% (33/107) of polymyositis (PM), 35.5% (33/93) of dermatomyositis (DM), 13.0% (3/23) of amyopathic DM, and 33.3% (1/3) of overlap myositis were positive for anti-ARS antibodies (Table 3). Among the 95 anti-ARS-positive IIM patients, 85 (89.4%) had IP, 54 (56.8%) arthralgia/arthritis, 24 (25.3%) had mechanic’s hand, 37 (38.9%) had high fever, and 31 (32.6%) had Raynaud’s phenomenon, which were consistent with previous reports [15]. The prevalence of these ASS symptoms was significantly higher in the anti-ARS-positive patients than in the negative patients (data not shown). There were seven anti-ARS-positive patients with other CTDs; two SSc patients were positive for anti-PL-12, two SLE patients were positive for anti-KS or anti-PL-12, and three RA patients were positive for anti-KS, anti-OJ or anti-PL-12.

**Table 1:** Comparison of the results between the new ELISA system and RNA-IP.

		RNA-IP		
		+	-	
anti-ARS ELISA	+	101*	1*	102
	-	0* (3)†	622* (619)†	622
	total	101* (104)†	623* (620)†	724

The results detecting the five anti-ARS antibodies (anti-Jo-1, PL-12, EJ, KS, and PL-7) are described (sensitivity: 100%, specificity: 99.8%).

Numbers in parenthesis are the results detecting all anti-ARS antibodies (including anti-OJ) (sensitivity: 97.1%, specificity: 99.8%).

**Table 2:** The frequency of each anti-ARS antibody in IIM, other CTD and IIP.

		RNA-IP(%)					
	ARS ELISA	Jo-1	PL-7	PL-12	EJ	KS	OJ
IIM	30.8% (77/250)	13.6	13.2	2.0	6.0	0.0	0.8
other CTDs	2.5% (7/276)	0.0	0.0	1.4	0.0	0.7	0.4
IIP	10.7% (18/168)	3.6	2.4	0.6	1.2	2.4	0.0
IPF	5.3% (2/38)	0.0	0.0	2.6	0.0	2.6	0.0
non-IPF	12.3% (16/130)	4.6	3.1	0.0	1.5	2.3	0.0

**Table 3:** The frequency of each anti-ARS antibody in subsets of IIM.

IIM classification	Total	Jo-1	PL-7	PL-12	EJ	KS	n (%)
I polymyositis	107	18	7	3	5	0	33 (30.8)
II dermatomyositis	93	13	10	1	9	0	33 (35.5)
III amyopathic dermatomyositis	23	0	2	0	1	0	3 (13.0)
IV malignancy-associated myositis	7	0	1	0	0	0	1 (14.3)
V juvenile myositis	1	0	0	0	0	0	0 (0)
VI overlap myositis	3	1	0	0	0	0	1 (33.3)
VII unclassified	6	2	3	1	0	0	6(37.5)

### Clinical significance of anti-ARS ELISA in IIP

Anti-ARS antibodies were positive in 10.7% (18/168) of IIP patients. Only two patients (5.6%) with IPF were positive for anti-ARS; conversely, 16 patients (12.1%) with non-IPF were positive for anti-ARS antibodies (Table 2). To investigate whether the anti-ARS-positive IIP were clinically distinct from anti-ARS-negative IIP patients, we compared clinical backgrounds and treatments between anti-ARS-positive and negative non-IPF patients (Table 4). The anti-ARS-positive patients were significantly younger and a higher proportion was female (p<0.01), and they were treated more frequently with glucocorticoids (GC) or the combination of GC and immunosuppressants (p<0.05 and p<0.01, respectively).

**Table 4:** Comparison of clinical backgrounds between anti-ARS (+) and (−) non-IPF patients.

	non-IPF n = 130		
	anti-ARS		
	(–) n = 114	(+) n = 16	p-value
age at the onset of the disease (yr) mean	69.6±9.5	56.9±14.5	<0.01
female (n; (ratio%))	39(34.2)	12(75.0)	<0.01
chronic (n; (ratio%))	104(91.2)	13(81.3)	N.S
subacute + acute (n; (ratio%))	5(4.4)	1(6.3)	N.S
acute (n; (ratio%))	2(1.8)	1(6.3)	N.S
glucocorticoids(GC) (n;(%))	49(43)	11(68.8)	<0.05
GC + immunosuppressants(IS) (n;(%))	19(16.7)	8(50.0)	<0.01
only drugs other than IS (n;(%))	8(7.0)	2(12.5)	N.S
PaO_2_ at rest (Torr) mean	75.9±14.9	86.5±37.4	N.S
SpO_2_ at rest (%) mean	95.7±2.4	97.1±2.1	<0.05
SpO_2_ after 6 min walk test (Torr) mean	88.6±5.5	86.9±6.0	N.S
%VC (%) mean	87.7±22.5	77.9±17.4	<0.05
%DLCO (%) mean	51.0±19.5	58.0±23.1	N.S
KL-6 (U/mL) mean	1132±949	1287±693	N.S
SP-D (ng/mL) mean	207±180	180±136	N.S

%VC: % vital capacity, %DLCO: % diffusing capacity of carbon monoxide.

## Discussion

Among MSAs/myositis-associated autoantibodies (MAAs), anti-ARSs are the most frequently detected (28–37% [1], [23], [24]) in adult IIM patients, and anti-ARS-positive patients develop common characteristic symptoms known as ASS. Not only IIM but also apparent IIP patients can be positive for anti-ARS antibodies because IP often precedes myositis [1], [14], [20], [25]. Both myopathy and IP anti-ARS-positive patients showed a better response to initial GC therapy but it can exacerbate the condition more often in anti-ARS-positive than in anti-ARS-negative patients [1], [26]. Therefore, anti-ARS antibodies are useful not only in diagnosis, predicting the clinical course and therapy decisions in IIM, but also in classifying IP patients and predicting late-onset myopathy in IP-preceding patients.

An immunoprecipitation assay has been used to detect each anti-ARS antibody but to date, it can only be performed in a limited number of laboratories. To detect them more easily and routinely, we aimed to establish an ELISA system using the six recombinant ARS antigens to simultaneously detect anti-Jo-1, PL-7, PL-12, EJ, OJ, and KS antibodies. We did not include anti-tyrosyl or phenylalanyl synthetase because they have been reported only in one case each. However, some differences in clinical manifestations and prognoses among patients expressing different ARS antibodies, especially between anti-Jo-1 and non-anti-Jo-1 patients, have been observed [14], [15]. However, different treatments for patients expressing different anti-ARSs have not been established. Currently, we treat anti-ARS-positive patients with expectation of a standard clinical course in which the disease can recur with tapering of GC and in which exacerbation of IP is associated with a poor prognosis [1], [14]. Therefore, presently, we are focusing on determining whether a patient with IIM or IIP is anti-ARS positive or not for the first screening when we begin treatment. This is why we decided to use a mixture of ARS antigens and not just single antigens to detect ‘multiple anti-ARS antibodies’ simultaneously.

We first prepared recombinant ARSs in *E. coli*, but recombinant PL-7 and OJ did not react well with their corresponding autoantibodies either using immunoblotting or an ELISA. For PL-7, structural conformation was important for antigenic activity because the recombinant PL-7 showed good reactivity only when it was expressed in a eukaryotic Hi-5 cell and was not denatured prior to being measured in the ELISA. Conversely, when recombinant PL-7 was denatured with urea or SDS, it was weakly detected with the PL-7 antibody, although its antigenicity was not completely lost. Such antigenic characteristics have also been reported previously by others [27]. This suggests that the synthetase epitope recognized by the anti-PL-7 antibody is in its native tertiary conformation.

In contrast, recombinant OJ (isoleucyl-tRNA synthetase) was not well detected even when it was expressed in Hi-5 cells and analyzed under non-denaturing conditions. This may be due to the unique feature of this isoleucyl-tRNA synthetase, which is a component of the multi-enzyme complex containing nine ARSs with three nonenzymatic factors [28], [29]. In screening tests, positivity of anti-OJ in patients’ sera was determined by the pattern of immunoprecipitation using HeLa cell extracts as originally described by Targoff *et al.* [28]. But there is a possibility that some ‘anti-OJ antibodies’ may recognize other components of the multi-enzyme complex rather than isoleucyl-tRNA synthetase itself, or alternatively the structural conformation of the complex may be important for recognition by anti-OJ, as was previously suggested by Targoff *et al.* [10]. They examined 11 patient sera with anti-OJ for evidence of reaction with other components of the complex. Ten out of 11 sera significantly inhibited enzyme activity of isoleucyl-tRNA synthetase, but some of them also significantly inhibited other ARSs such as leucyl-, lysyl-, or arginyl-tRNA synthetases. Moreover, immunoblot analysis of anti-OJ revealed that the majority of the sera could not identify a shared band and only a few sera recognized isoleucyl-tRNA synthetase. These results suggest that most ‘anti-OJ sera’ may react with multiple synthetases of the multi-enzyme complex or react with conformational epitopes of the complex. For this reason, we considered that it would be difficult to prepare the immunoreactive OJ antigen as a single molecule; therefore, we developed an ELISA system using the other five recombinant ARSs. This may not significantly affect the sensitivity of the ELISA because the prevalence of anti-OJ antibodies in patients is very low among the six anti-ARS antibodies.

The efficiency of this newly established ELISA system was acceptable because the sensitivity and specificity of the system compared with RNA immunoprecipitation were 97.1% and 99.8%, respectively, even if anti-OJ-positive sera was not excluded. The prevalence of anti-ARS in our IIM cohort was comparable with previous reports [1], [2]. It was noteworthy that 10.7% of IIP patients, and in particular, 12.1% of non-IPF patients were positive for anti-ARS antibodies and there were some differences between anti-ARS-positive and negative IIP patients in their clinical backgrounds and treatments. Anti-ARS-positive patients were treated significantly more frequently with GC or the combination of GC and immunosuppressants. However, we are not yet ready to recommend immunosuppressive therapy for anti-ARS-positive IIP patients because we have not yet collected enough data on their clinical response and prognosis. Although some of these anti-ARS-positive IIP patients might develop myopathy later, it suggests that the measurement of anti-ARS antibodies may be useful in stratifying patients into disease subsets, which may help in predicting their clinical course.

A line-blot assay for the detection of multiple MSAs/MAAs (EUROLINE Myositis Profile 3) has been used in which anti-Jo-1, PL-7, PL-12, EJ, and OJ are included. This system can detect and discriminate MSAs/MAAs without further anti-ARS tests, but it does not include anti-KS, which has a stronger association with IIP than myositis [30]. To address this point, our system can more efficiently detect anti-ARS and therefore, is the preferred assay to use for IIP patients than the line-blot assay, although our ELISA does not aim to discriminate specificity for each anti-ARS antibody.

In conclusion, our ELISA system using a mixture of five recombinant ARSs shows similar efficiency to RNA immunoprecipitation and makes it possible to more readily detect anti-ARS antibodies in patients with PM/DM and IIP, and can be widely applied in daily practice.

## Supporting Information

Table S1 **The list of approval by institutional review boards of all participating centers.**
(XLSX)
